# Kirenol alleviates diabetic nephropathy via regulating TGF-β/Smads and the NF-κB signal pathway

**DOI:** 10.1080/13880209.2022.2112239

**Published:** 2022-09-08

**Authors:** Jialin Li, Jiawen Zhang, Meng Yang, Xiaocui Huang, Meng Zhang, Xiansong Fang, Suzhen Wu

**Affiliations:** a Key Laboratory of Prevention and Treatment of Cardiovascular and Cerebrovascular Diseases, Ministry of Education, Gannan Medical University, Ganzhou, China; b School of Pharmacy, Gannan Medical University, Ganzhou, China; c School of Basic Medicine, Gannan Medical University, Ganzhou, China; d First Affiliated Hospital, Gannan Medical University, Ganzhou, China

**Keywords:** TNF-α, IL-6, mesangial cells, fibronectin, collagen IV

## Abstract

**Context:**

Kirenol possesses anti-inflammatory, antifibrotic and anti-arthritic effects. However, its reno-protective effects against diabetic nephropathy (DN) have not been evaluated.

**Objective:**

This study explores the reno-protective effects of kirenol against DN and clarifies the potential mechanisms.

**Materials and methods:**

The mesangial cells were treated with 20 µM kirenol and 10 ng/mL human recombinant TGF-β1 or 30 mM glucose for 24 h. Then the cells were harvested to assay the expression of the target genes or proteins. Thirty C57BL/6J male mice were given high-fat diet with streptozotocin injection to induce diabetes and then were randomized into three groups (*n* = 10): vehicle administration (DM group), 2 mg/kg kirenol (DM + kirenol group) and 200 mg/kg metformin (Met group) for 3 months, orally. A healthy group (Con, *n* = 10) was included as the control.

**Results:**

Compared to the DM group, kirenol treatment decreased the phosphorylation of Smad2/3 and NF-κB (0.64- and 0.43-fold) as well as the accumulation of FN and Col IV (0.58- and 0.35-fold); moreover, the expression of IκBα was restored to normal level by kirenol treatment both *in vivo* and *in vitro*. After kirenol treatment, IL-6 expression was decreased 0.35- and 0.57-fold, and TNF-α expression was decreased 0.34- and 0.46-fold, *in vitro* and *in vivo*, respectively. Furthermore, kirenol alleviated the glomerular basement membrane thickness and foot process fusion.

**Discussion and conclusions:**

Kirenol could alleviate DN by downregulating the TGF-β/Smads and the NF-κB signal pathway. Our study provides a potential mechanism for the treatment of DN with kirenol.

## Introduction

Diabetes mellitus (DM) is a worldwide epidemic disease characterized by persistent hyperglycaemia. Long-term high blood glucose level is associated with multiple complications, such as diabetic nephropathy (DN), diabetic retinopathy and diabetic cardiomyopathy. DN is among the most serious complications of DM (Jin et al. 2015). The overexpression and the excessive accumulation of extracellular matrix protein (ECM) such as fibronectin (FN) and collagen IV (Col IV) in mesangial cells and the glomerular basement membrane thickening are the hallmarks of DN (Kanwar et al. 2011; Zeng et al. 2019). Commercially available drugs for curing DN have not been developed; the main alternative medicines include metformin, sulfonylureas, α-glucosidase inhibitors and DPP-4 inhibitors, but these antidiabetic drugs cause many side effects such as hypoglycaemia, liver damage, lactic acidosis and diarrhoea to patients (Liang et al. 2018). Preclinical studies have shown that extracts from natural herbal medicine for the treatment of diabetes-related complications have a few side effects (Tahrani et al. 2016; Tang et al. 2018). Kirenol, which is isolated from the plants *Siegesbeckia pubescens* Makino, *Siegesbeckia orientalis* L. and *Siegesbeckia glabrescens* Makino (Asteraceae), is the major active diterpenoid component, which possesses anti-inflammatory, antifibrosis and anti-arthritic effects (Wu et al. 2019; Ren et al. 2020; Quan et al. 2021). However, its reno-protective effects and the potential molecular mechanism have not been evaluated.

The TGF-β/Smads signalling pathway is a classic signalling pathway for DN and glomerulosclerosis (Li et al. 2003; Meng et al. 2016; Chen et al. 2017). The downstream signalling molecules include Smad2, Smad3, Smad4 and Smad7. The TGF-β/Smads signalling pathway is activated both in the DN animal model and high glucose-induced diabetic cell model, and then facilitates p-Smad2, p-Smad3 and Smad4 to form a complex translocated to the nucleus as a transcriptional factor of ECM (Xin et al. 2013; Guan et al. 2018). This phenomenon leads to the overexpression and deposition of ECM and the development of DN. Whether kirenol can improve DN through this pathway has not been reported.

Nuclear transcriptional factor NF-κB can mediate inflammation through various signal transduction pathways (Zou et al. 2018; Zeng et al. 2020). NF-κB is mainly distributed in the glomerulus, renal interstitial and epithelial cells of renal tubules (Zhang et al. 2020). It is closely related to immune response, production of inflammatory factors, tumour occurrence, cell proliferation and apoptosis. The NF-κB signalling pathway is one of the main pathways in the development of DN (Zheng et al. 2020). NF-κB usually binds to its inhibitory protein IкBα and stays in the cytoplasm inactively (Yu et al. 2020). IκBα is then phosphorylated at Ser 32 and 36 after undergoing TLR4, IL-6 and TNF-α stimulation, causing the proteasome-dependent degradation of IкBα, exposing the nuclear localization signal of NF-κB, inducing the translocation of NF-κB from the cytoplasm to the nucleus, and finally promoting the expression of IL-6 and TNF-α. The NF-κB signalling pathway is continuously activated in DN, resulting in high expression of the downstream inflammatory factors, thereby inducing the inflammatory response and promoting the development of DN (Donate-Correa et al. 2020; Möller-Hackbarth et al. 2021). Kirenol has inhibitory effects on the NF-κB signalling pathway in diabetic cardiomyopathy and reduces inflammation (Wu et al. 2019). It was also reported to have anti-inflammatory and wound healing effects in diabetic rats (Ren et al. 2020; Ibrahim et al. 2021). However, whether kirenol could inhibit the NF-κB signalling pathway and reduce inflammation in DN has not been investigated.

In this study, we investigated the reno-protective effects of kirenol against DN and clarified the potential mechanisms in both diabetic animals and cell models.

## Materials and methods

### Cell culture and treatment

Primary mesangial cells (MCs) from mouse was obtained from Procell company and cultured in DMEM (Solarbio, #31600) supplemented with 20% foetal bovine serum (Biological Industries, #1928625), 100 units/mL penicillin, and 100 μg/mL streptomycin at 37 °C under 5% CO_2_ atmosphere. MCs in all groups were cultured with normal glucose (5.5 mM, Sigma, #SLCC4951) containing DMEM and then treated as follows: Nontreated MCs were named Con group; MCs treated with 30 mM glucose for 24 h were named HG group; MCs treated with 10 ng/mL human recombinant TGF-β1 (R&D Systems, Minneapolis, #240-B) for 24 h were named TGF-β1 group; MCs pre-treated with 20 µM kirenol (purchased from Chengdu Push Biotechnology Co., Ltd., PS011133) for 30 min, and then cotreated with 10 ng/mL TGF-β1 for 24 h were named TGF-β1+ kirenol group; MCs pre-treated with 20 µM kirenol for 30 min, and then cotreated with 30 mM glucose were named HG + kirenol group; MCs treated with 20 µM kirenol were named NG + kirenol group. After that, the cells were harvested to assay the expression of the target genes or proteins. Each *in vitro* experiment was repeated three times.

### Animal experiments

C57BL/6J male mice (weight: approximately 20 g) supplied by the Slac King Laboratory Animals Co. Ltd. (License No. SCXK2016-0002). All the animal experiments were approved by the ethics committee of Gannan Medical University (2020027) and adhered to the Animal Care and Use Committee of Gannan Medical University guidelines (License No. SYXK2018-0004). After adapting to the environment for a week, the mice were randomly divided into two groups. The mice in one group were fed a high-fat diet (21.4% fat, 43.1% carbohydrate and 21.2% protein with a total caloric value of 46 kJ) for 2 months, after which 100 mg/kg STZ (Sigma Chemicals Co., Ltd., USA) dissolved in sodium citrate buffer at pH 4.2–4.5 was intraperitoneally injected for 5 consecutive days after 12 h of fasting (no fasting from water). The random blood glucose was tested 2 weeks later after the last administration of STZ, the mice with random blood glucose ≥16.7 mmol/L were considered diabetic mice. Mice in the control group were fed with a regular diet and injected equal volume of citrate buffer as solvent control (*n* = 10). Then, the diabetic mice were treated with an equal volume of normal saline (DM group), 2 mg/kg kirenol (DM + kirenol group) and 200 mg/kg metformin (Met group) as a positive control, *n* = 10/group. The dosages of kirenol and metformin are set as previously described (Wang et al. 2018; Zou et al. 2021). The administration was stopped 3 months later. Then, the animals were sacrificed, and the kidneys were immediately removed for the following experiments.

### Protein extraction and Western blot analysis

The MCs were harvested and lysed with RIPA buffer after being treated with kirenol as indicated concentration and time. The total protein concentration was tested by a BCA protein assay kit (Thermo Scientific, WK337199) and the protein was separated by SDS-PAGE. Briefly, after transferring proteins from the gels to polyvinylidene difluoride membranes (Merck Millipore, R1NB76879), the membranes were blocked with 5% skimmed milk, and then incubated with the following primary antibodies: fibronectin (1:10,000, Merck Millipore, #AB1954), p-Smad2/3 and Smad2/3 (1:1000, Cell Signalling Technology, 8828S and 8685S), β-actin (1:1000, Cell Signalling Technology, 3700S), Col IV (1:1000, Proteintech, #19797-1-AP), NF-κB, p-NF-κB and IκBα (1:1000, Cell Signalling Technology, 4764S, 3033S and 4814S) at 4 °C overnight. Afterward, the membranes were incubated with horseradish-conjugated secondary antibodies at room temperature for 2 h. Finally, immunoblots were developed using enhanced chemiluminescence and visualized with Bio-Rad Chemi-Doc MP or Amersham Imager 600 imaging system. The densities of the bands were quantified using ImageJ software.

### Real-time quantitative polymerase chain reaction (RT-qPCR)

TRIzol reagent (Ambion, #152207) was used to extract the RNA from MCs and the renal tissue of C57 BL/6J mice according to the manufacturer’s protocol. Reverse transcription, DNA replication and the primer sequences are described in our previous publication (Li J, Wu B et al. 2020).

### Cell viability assay

Cell viability was assayed using the MTT method. Approximately 1 × 10^5^ cells/well were plated in a 96-well plate. The next day, cells were treated with the indicated concentration of kirenol for 24 h, incubated with 5 mg/mL MTT solution for another 4 h, and then added with DMSO to dissolve the formazan. Thereafter, the absorbance was assayed at 570 and 690 nm. Then, the cell viability was calculated by normalizing to DMSO control with GraphPad Prism 6 software.

### Electrophoretic mobility shift assay (EMSA)

The nucleoproteins were extracted with a nucleus protein extract kit (Beyotime Biotechnology, #P0033). The nucleoproteins were incubated with the biotin-labelled double-stranded NF-κB DNA probes (Beyotime Biotechnology, #GS056A) for 30 min at room temperature, resolved on a 4% polyacrylamide gel in 0.5 × TBE, and then transferred with nylon membrane at 380 mA for 45 min at 4 °C. Thereafter, the nylon membrane was cross-linked with a 254 nm UV lamp. Finally, streptavidin-HRP conjugated chemiluminescence reagents (Beyotime Biotechnology, #A0303) were added to the nylon membrane, and bands were visualized using a Bio-Rad Chemi-Doc MP imaging system. The sequences of the NF-κB consensus oligo are as follows: 5′-AGTTGAGGGGACTTTCCCAGGC-3′ and 3′-TCAACTCCCCTGAAAGGGTCCG-5′.

### Histological analysis

Renal samples were fixed and embedded as described in our previous publication for morphological experiments (Li J, Xie F et al. 2020). The samples were cut into 4 µm thick slices and then mounted on glass slides. The fixed sections were stained with haematoxylin and eosin (H&E) and periodic acid-Schiff (PAS) reagent to observe the renal morphological changes. The deposition of collagen was demonstrated by Masson’s trichrome staining. Finally, the morphological changes in the slides were observed under a light microscope, and three random photos were randomly selected to analyze the morphological changes with Image-J.

### Immunohistochemistry (IHC)

Paraffin (4 µm) sections of the renal cortex were deparaffinized in dimethylbenzene, rehydrated in graded ethanol, and then washed with 0.01 M PBS. Antigen retrieval was performed by dipping slides into the citrate buffer solution heated with a microwave. The sections were incubated with 3% H_2_O_2_ to quench the endogenous peroxidase. Subsequently, the slides were blocked with 3% BSA, and then incubated with the following primary antibodies: fibronectin (1:200, BD Biosciences), Col IV primary antibody (1:100, Proteintech) overnight at 4 °C, and then incubated with the corresponding secondary antibody. Thereafter, the slides were stained with a 3,3′-diaminobenzidine solution. Then, the immunoblots in the slides were examined under a light microscope (Olympus, Japan), and the expression of target proteins was examined with ImageJ software.

### Transmission electron microscopy

The ultrastructural changes in the renal cortex were observed under a transmission electron microscope (JEOL, Japan). Briefly, the renal samples (about 1 mm^3^ pieces) were prefixed in 2.5% glutaraldehyde for 2 h and then changed to 1% osmium tetroxide for another 2 h. Then, the samples were dehydrated with gradient alcohol and embedded in epoxy resin. The samples were then examined under a transmission electron microscope, and three random glomerular transmission electron micrographs at 10,000× magnification was obtained to determine the renal injury.

### Statistical analysis

Data presented as mean ± SEM. The statistical significance among groups was analyzed by multiple comparisons test under One-way ANOVA. The statistical analysis was performed using GraphPad. *p*-Value <0.05 was considered significant.

## Results

### Kirenol could downregulate HG/TGF-β induced the TGF-β/Smads signalling pathway and ECM expression in mesangial cells

**Figure 1.**
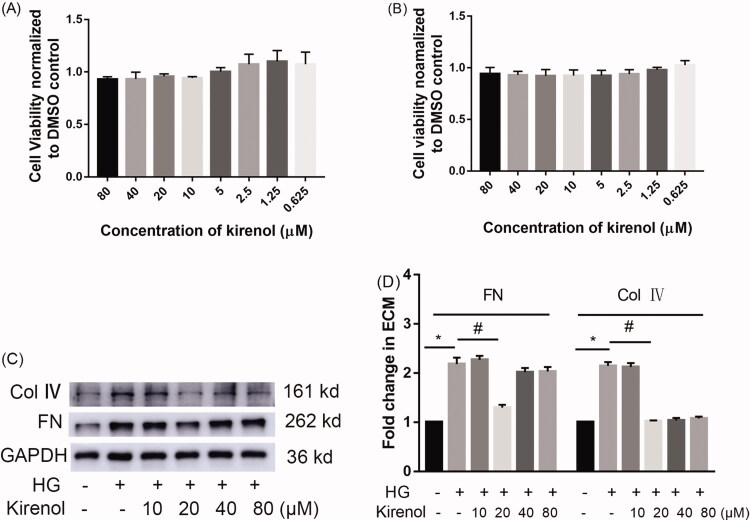
Effects of kirenol on mesangial cells (A) and podocytes (B) were tested by MTT, the dosage range of 0.625–80 μM kirenol did not remarkably inhibit the growth of mesangial cells and podocytes. The dose-response effects of kirenol on the expression of FN and Col IV were detected by Western blot (C) and (D) the corresponding relative expression of FN and Col IV calculated by Image J. Data are expressed as mean ± SEM. *p < 0.05, compared with the normal control group; #*p* < 0.05, compared with the HG treated group.

First, we performed MTT to determine a relatively safe dosage for mesangial cells. The result indicated that 0.625–80 μM kirenol （IC_50_ = 0.29 mM）did not inhibit the growth of mesangial cells (Figure 1(A)). Podocyte apoptosis also plays an important role in the development of DN, therefore we performed MTT to detect the effects of kirenol on the cell viability of podocytes and the result also indicated that 0.625–80 μM kirenol did not obviously affect the cell viability (Figure 1(B)). The dose-response experiment showed that 20 μM kirenol could effectively inhibit the deposition of ECM (Figure 1(C,D)). Accordingly, we set 20 μM kirenol for the treatment of cells in the following experiments.

**Figure 2.**
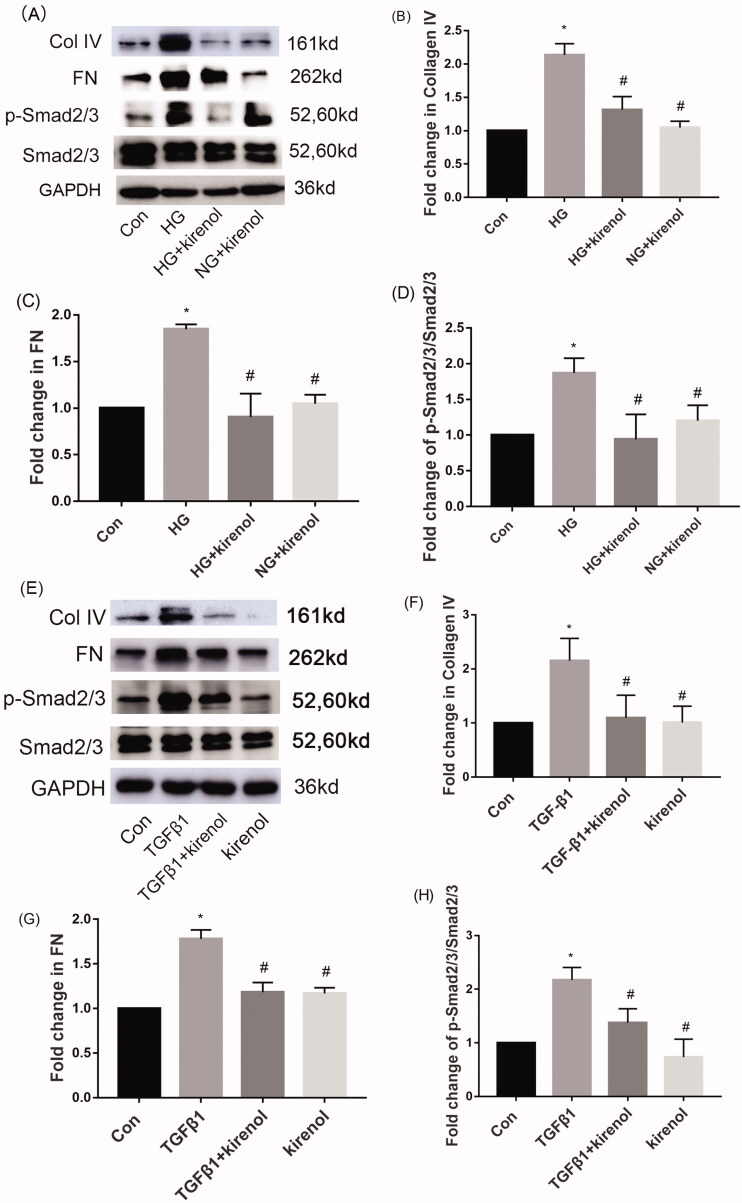
Kirenol downregulated HG/TGF-β-induced TGF-β/Smads signalling pathway activation and ECM expression in mesangial cells. (A) Protein levels of FN, Col IV, Smad2/3 and p-Smad2/3 induced by HG were detected by Western blot analysis. (B–D) Relative protein expression analysis. (E) Protein levels of FN, Col IV, Smad2/3 and p-Smad2/3 induced by TGF-β1 were detected by Western blot analysis. (F–H) Relative protein expression analysis. Data are expressed as mean ± SEM. *p < 0.05, compared with the normal control group; #*p* < 0.05, compared with the HG group or TGF-β1 group.

The TGF-β/Smads signal pathway is activated in both mesangial cells induced by HG/TGF-β and in animal models of diabetic nephropathy, causing the accumulation of ECM, which eventually leads to DN. Our research found that kirenol could inhibit the activation of the TGF-β/Smads signalling pathway induced by HG, and then suppress the expression of ECM (Figure 2(A–D)). Moreover, kirenol could also inhibit TGF-β-induced phosphorylation of Smad2/3 and then attenuate the deposition of ECM (Figure 2(E–H)). These results suggest that kirenol may improve diabetic nephropathy by inhibiting the TGF-β/Smads signalling pathway.

### Kirenol could reduce HG-induced inflammation by inhibiting activation of the NF-κB signalling pathway in mesangial cells

**Figure 3.**
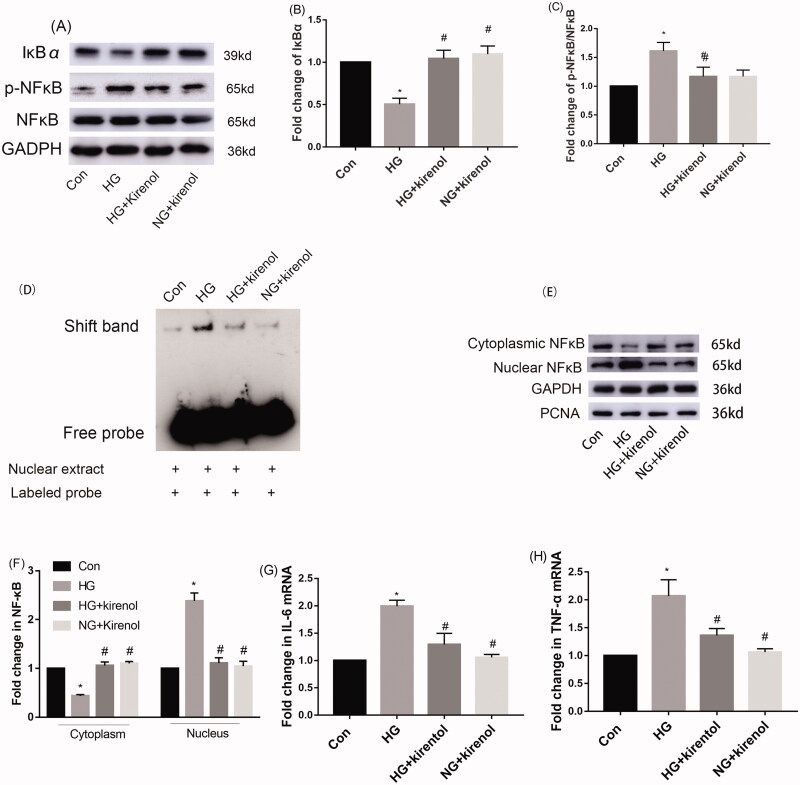
Kirenol inhibited the phosphorylation of NF-κB and promoted the expression of IκBα in HG-induced mesangial cells. (A) Protein levels of NF-κB, p-NF-κB and IκBα induced by HG were detected by Western blot analysis. (B, C) Relative protein expression analysis. (D) The DNA binding activity of NF-κB in mesangial cells was detected by EMSA. (E) The translocation of NF-κB from the cytoplasm to the nucleus in the mesangial cells was tested by separating the cytoplastic protein and nucleus protein and then determined by Western blot analysis and (F) the corresponding relative protein expression analysis. The gene expression levels of IL-6 (G) and TNF-α (H) were tested by RT-qPCR. Data are expressed as mean ± SEM. *p < 0.05, compared with the normal control group; #*p* < 0.05, compared with the HG group.

Diabetes is also an inflammatory disease. Diabetic nephropathy is accompanied by inflammation. NF-κB is a transcriptional factor of IL-6 and TNF-α, the phosphorylation and translocation of NF-κB will drive the expression of inflammatory factors such as IL-6 and TNF-α. Our study found that kirenol could inhibit the HG-induced phosphorylation of NF-κB. Moreover, IκBα, which is a negative regulator of NF-κB signalling pathway, was significantly increased by kirenol treatment (Figure 3(A–C)). We designed an NF-κB probe that binds to nuclear proteins and then performed EMSA to investigate their binding activities. The EMSA results showed that the binding activity of the NF-κB probes to the nuclear protein in HG-induced mesangial cells was remarkably suppressed by kirenol treatment (Figure 3(D)). These results indicated that kirenol treatment markedly inhibited the HG-induced NF-κB signalling pathway in mesangial cells.

Furthermore, the cytosolic and nuclear proteins were separated to investigate the distribution of NF-κB in the cytoplasm and nucleus by Western blot analysis. The results indicated that kirenol could inhibit the translocation of NF-κB from the cytoplasm to the nucleus (Figure 3(E,F)).

The gene expression of IL-6 and TNF-α, which are the downstream target gene of the NF-κB signal pathway was tested by real-time quantitative PCR. The results showed that HG-induced expressions of IL-6 and TNF-α were inhibited by kirenol treatment (Figure 3(G,H)).

### Effects of kirenol on renal morphology (injury) and biochemical indicators of diabetic mice

**Figure 4.**
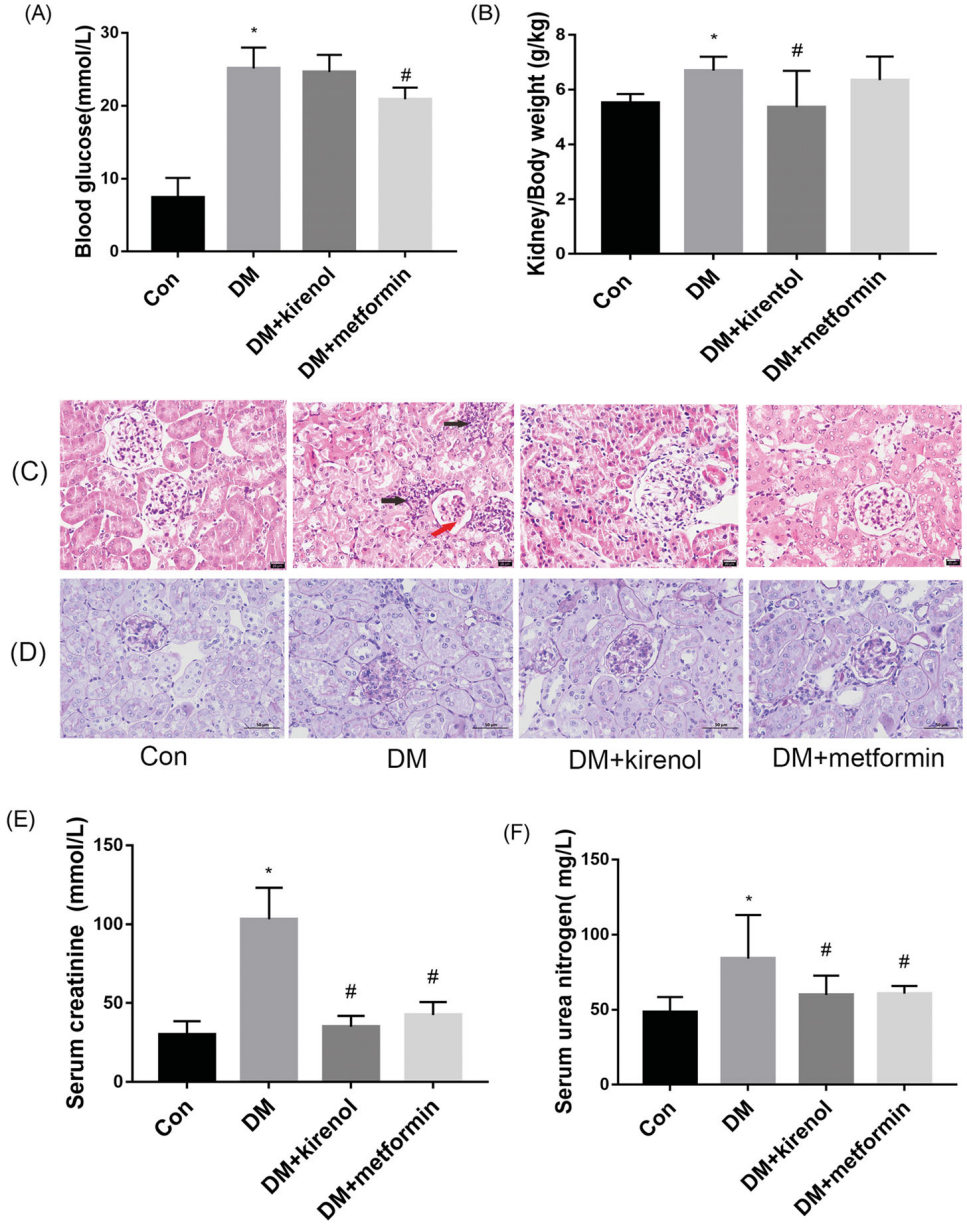
Kirenol could not reduce the blood glucose level of the diabetic mice, but it improved the renal damage of diabetic mice. (A) Blood glucose level of the mice. (B) Kidney/body weight ratios. (C) Renal pathological changes were analyzed by haematoxylin and eosin (H&E) staining and (D) periodic-acid Schiff (PAS) staining. Red arrow showed the mesangial expansion and the black arrows showed the focal interstitial inflammation. The content of serum creatinine (E) and urea nitrogen (F) was tested by using the commercially available kits. *p < 0.05 vs. control group, #*p* < 0.05 vs. DM group.

To verify whether kirenol can also improve diabetic nephropathy at animal levels, we established a diabetic mouse model and then treated them with kirenol. Three months after the administration, the mice were sacrificed, and their renal cortex and serum were obtained for the following experiment. Compared to the DM group, metformin treatment lowered blood glucose and improved the kidney/body weight ratio, however, kirenol treatment could not lower the blood glucose but improved the kidney/body weight ratio, as shown in Figure 4(A,B). Both kirenol and metformin could ameliorate the serum creatinine (Scr) and blood urea nitrogen (BUN), as shown in Figure 4(E,F). The inconsistent effect of metformin on kidney/body weight ratio among serum creatinine and urea nitrogen may be because serum creatinine and urea nitrogen are more sensitive than kidney/body weight ratio and/or the molecular mechanism of the reno-protective effects are different. H&E staining demonstrated that kirenol and metformin could alleviate diabetes-induced mesangial expansion and focal interstitial inflammation and glomerular atrophy, as shown in Figure 4(C). PAS staining showed that kirenol and metformin could alleviate glomerular sclerosis and the collapse of the capillary loop in the glomerular of diabetic mice, as shown in Figure 4(D).

### TEM showed that kirenol could improve GBM thickness and podocyte failure

**Figure 5.**
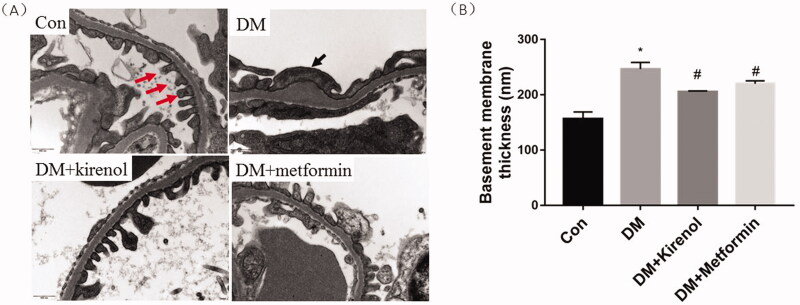
Kirenol could improve GBM thickness and podocyte failure. (A) TEM showed that kirenol alleviated diabetes-induced foot process fusion and podocytes diminish. Red arrows indicate foot process and black arrow indicate foot process effacement. (B) Quantitative analysis of GBM thickness (nm). Data are expressed as mean ± SEM. *p < 0.05 vs. control group, #*p* < 0.05 vs. DM group.

The results in Figure 5 showed that kirenol and metformin could alleviate the GBM thickness of diabetic mice. The shapes of the podocytes in kirenol- and metformin-treated groups were more regular than those of diabetic mice. The foot process fused together along the GBM surface, and some of the podocytes disappeared in the glomerulus of the diabetic mice. Kirenol treatment alleviated the GBM thickening and foot process effacement.

### Kirenol could inhibit TGF-β/Smads signal pathway and attenuate ECM accumulation in the kidneys of diabetic mice

**Figure 6.**
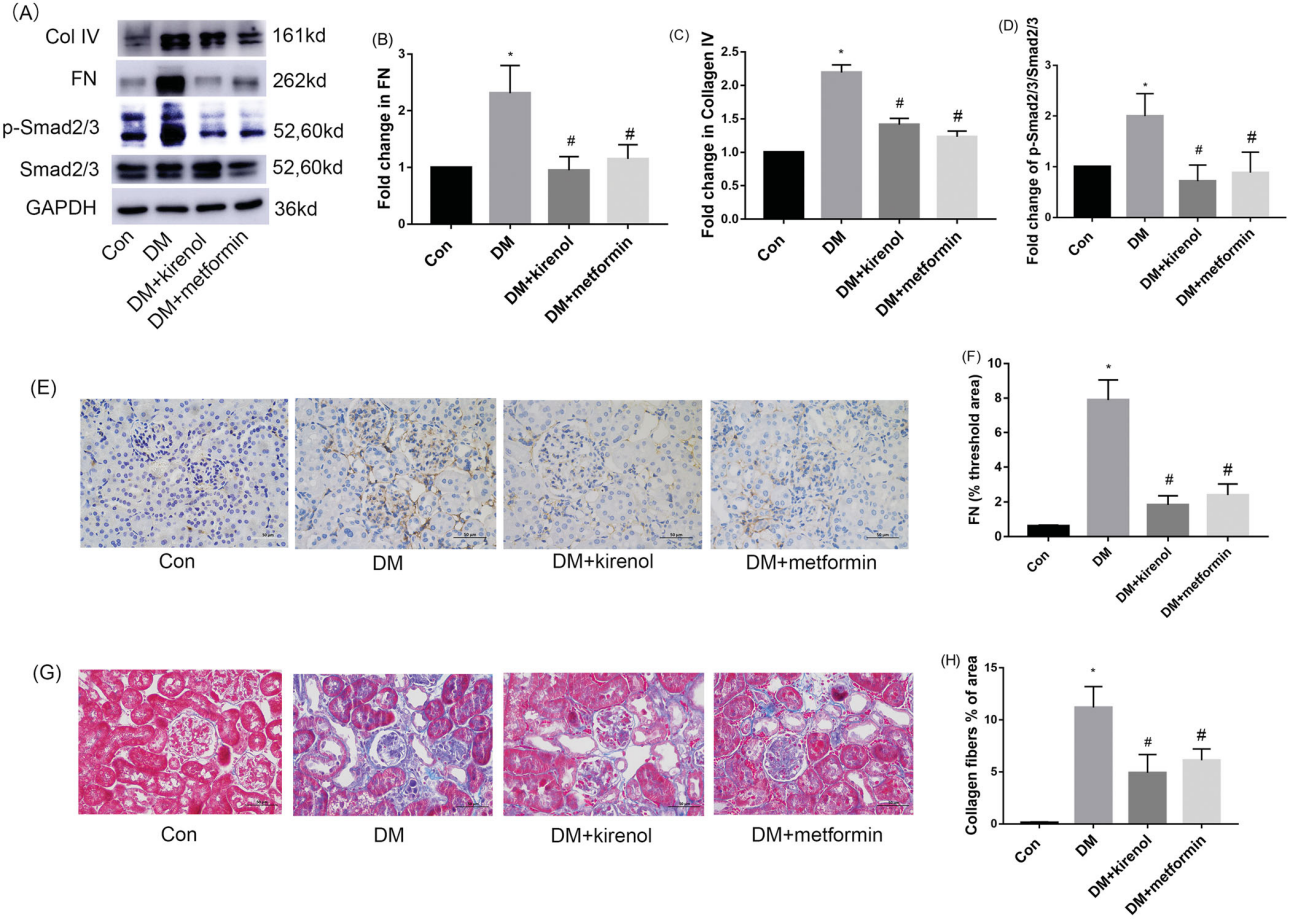
Kirenol inhibited the TGF-β/Smads signal pathway and attenuated ECM accumulation in the kidneys of diabetic mice. (A) The protein expression of FN, Col IV, Smad2/3 and p-Smad2/3 was detected by Western blot analysis. (B–D) Corresponding relative protein expression analysis of FN, Col IV and p-Smad2/3. (E) The protein expression of FN was detected by IHC. (C) The percentage of FN positive areas was determined using Image-J software. (G) Collagen fibres accumulation in the renal cortex was detected by Masson staining. Magnification: 400×, Scale bars: 50 μm. (H) The percentage of collagen fibre positive areas were determined by Image-J software. Data are expressed as mean ± SEM. *p < 0.05 vs. control group, #*p* < 0.05 vs. DM group.

Excessive deposition of ECM such as FN and Col IV in the glomerulus is a key marker of progressive glomerulosclerosis and eventually causes kidney hypertrophy, GBM thickening and podocyte impairment. After kirenol treatment of diabetic mice for 3 months, the expression of FN and Col IV in the renal cortex was detected by Western blot, the results indicated that kirenol could suppress the diabetes-induced expression of FN and Col IV by inhibiting the TGF-β/Smads signal pathway, as shown in Figure 6(A–D). The IHC results also showed that kirenol could alleviate the expression of FN in the kidney of diabetic mice, as shown in Figure 6(E,F). Masson staining also indicated more collagen fibre accumulation in the kidney of diabetic mice compared with that of the kirenol-treated group, as shown in Figure 6(G,H). Therefore, kirenol could inhibit the TGF-β/Smads signal pathway and attenuate ECM accumulation in the kidneys of diabetic mice.

### Kirenol could inhibit the NF-κB signal pathway and attenuate inflammation in the kidneys of diabetic mice

**Figure 7.**
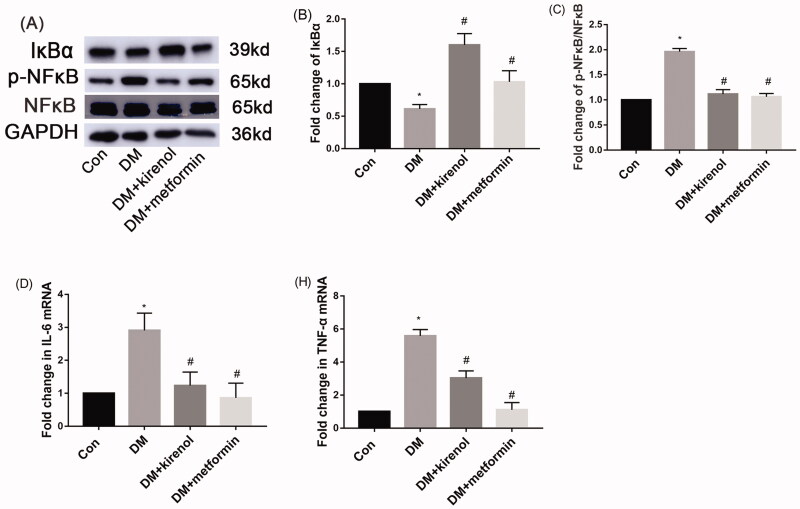
Kirenol could inhibit the NF-κB signal pathway and attenuate inflammation in the kidneys of diabetic mice. (A) The protein expression of IκBα, NF-κB and p-NF-κB was detected by Western blot analysis. (B, C) Corresponding relative protein expression analysis. The gene expression of IL-6 (D) and TNF-α (E) tested by RT-qPCR. Data are expressed as mean ± SEM. *p < 0.05 vs. control group, #*p* < 0.05 vs. DM group.

After 3 months of treatment with kirenol, Western blot and RT-qPCR were performed to detect whether the kidney inflammation of diabetic mice was alleviated by kirenol. Figure 7(A–C) indicated that kirenol could inhibit the phosphorylation of NF-κB in the kidneys of diabetic mice. Moreover, IκBα, which is a negative regulator of the NF-κB signal pathway, was downregulated in the kidneys of diabetic mice, and kirenol treatment upregulated the expression of IκBα, also as shown in Figure 7(A–C). Furthermore, the gene expression of IL-6 and TNF-α increased in the kidney of diabetic mice, and administration of kirenol significantly inhibited the gene expression levels of IL-6 and TNF-α, as shown in Figure 7(D,E).

**Figure 8.**
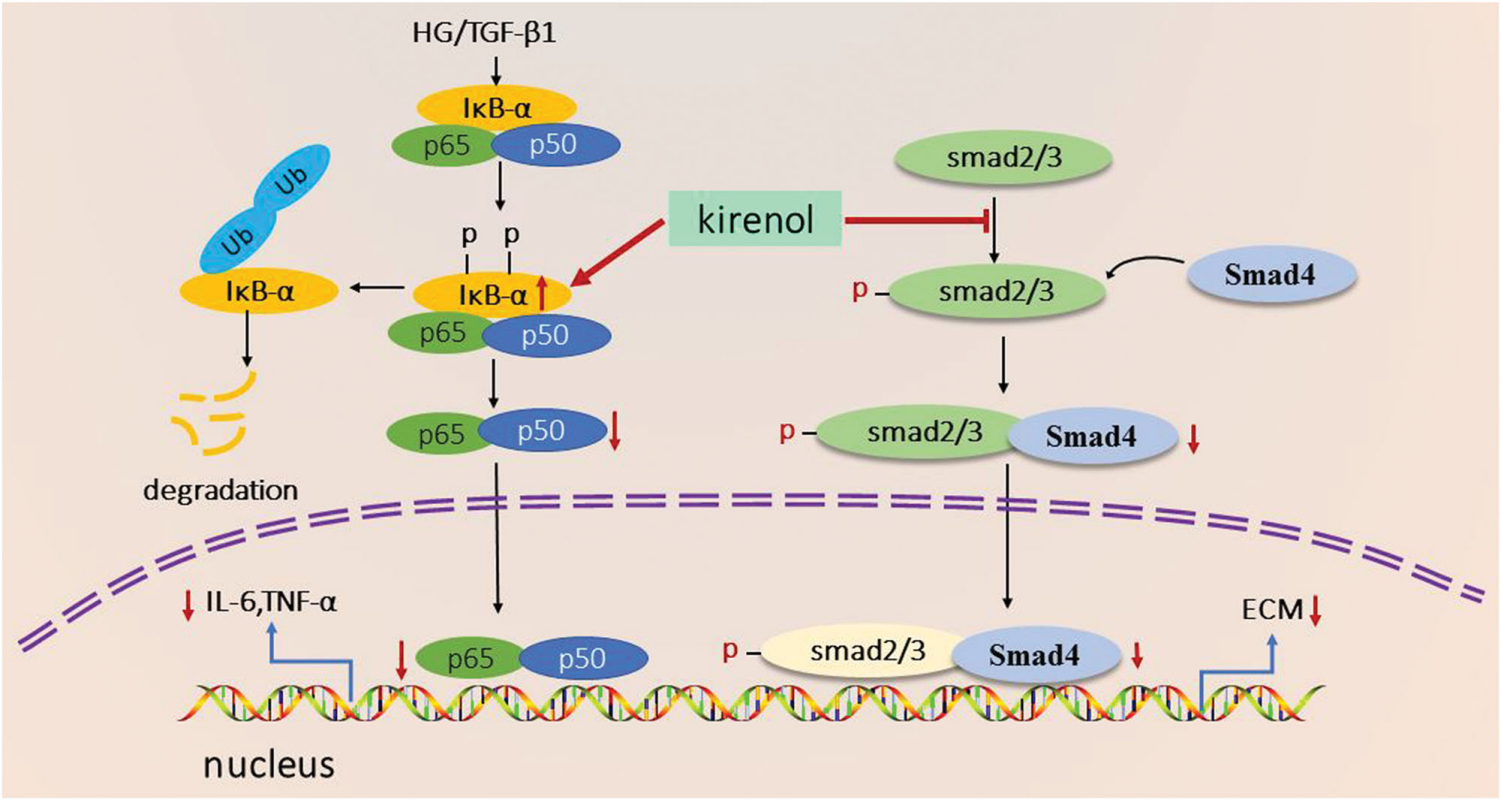
Proposed mechanism of the reno-protective effects of kirenol. Kirenol could increase the protein level of IκBα, which was a negative regulator of the NF-κB signal pathway. Kirenol could also negatively regulate the TGF-β/Smads signal pathway, finally exerting reno-protective effects.

Taken together, kirenol treatment may attenuate reno-fibrosis and reno-inflammation by inhibiting TGF-β/Smads and NF-κB signal pathway as summarised in Figure 8.

## Discussion

DN is one of the most common chronic kidney diseases caused by hyperglycaemia, both type 1 and type 2 diabetes will eventually progress to DN (Tramonti and Kanwar 2011; Zhou et al. 2014; Juin et al. 2021). The levels of serum creatinine and urea nitrogen, and the thickness of GBM were reported to be increased both in diabetic human beings and in the diabetic animal model, moreover, the kidney/body weight ratio which is a renal hypertrophy index also been reported to be elevated in the diabetic animal model (Nielsen et al. 2009; Qian et al. 2016). The current study, for the first time, showed that kirenol could not downregulate the blood glucose level of diabetic mice. However, it could alleviate the kidney/body weight ratio, diabetes-induced GBM thickening, the kidney damage indicators such as kidney/body weight ratio, and GBM thickening; serum creatinine and urea nitrogen were also improved by kirenol treatment.

The main pathological feature of DN is glomerular sclerosis, which is manifested by the hypertrophy of glomerular mesangial cells, and overexpression of ECM, including collagen, fibronectin and laminin; these proteins are secreted outside the mesangial cells and deposited in the glomerulus, leading to glomerular sclerosis, which eventually develops into renal fibrosis (Hua et al. 2001; Zhang et al. 2012). In this study, we found the expression of FN and Col IV was increased both in the HG-induced diabetic cell model and STZ-induced diabetic animal model. However, this study, for the first time, found that the overexpression of ECM induced by HG/STZ could be attenuated by the treatment of kirenol.

The TGF-β/Smads signal pathway is widely known to be activated in diabetic kidney disease, and the persistent activation of this pathway will lead to renal fibrosis (Avila-Carrasco et al. 2019). Both TGF-β and HG could activate the TGF-β/Smads signal pathway and promote the excess expression of ECM, which was a hallmark of DN (Li J, Wu B et al. 2020). Smad2/3 and Smad4 are downstream factors of the TGF-β/Smads signal pathway, followed by the stimulation of HG or TGF-β, Smad4 will bind to the phosphorylated Smad2/3 to form a complex and then translocated to the nucleus as transcriptional factors to promote the expression of ECM and inhibition of TGF-β/Smads signal pathway could alleviate excess ECM expression and improve DN (Hu et al. 2021). Consistent with these studies, our work indicated that kirenol could inhibit the TGF-β/Smads signal pathway and then alleviate ECM accumulation both in DN animal and cell models.

DN is also driven by inflammation which was widely accepted as one of the most crucial pathogenic factors for DN. The secretion of cytokines such as TNFα and IL6 will aggravate DN-related inflammation (Li M et al. 2020). The activation of NF-κB will lead to the expression of TNFα and IL6. In the diabetic kidney, HG-induced phosphorylation and the subsequent translocation of NF-κB from the cytoplasm to the nucleus will trigger inflammation, and subsequently, produce excessive TNFα and IL6 (Aladaileh et al. 2021). IκBα is a negative regulator of the NF-κB signal pathway. IκBα binds to NF-κB in the cytoplasm and then inhibits the nucleus translocation of NF-κB under normal circumstances, and finally inhibits the expression of inflammatory factors, such as IL-6 and TNF-α (Sureshbabu et al. 2016). Multiple drugs targeting inflammatory pathways have also been used for antidiabetic cardiometabolic diseases and other diabetic complications. A study analyzed the fasting blood glucose and serum levels of TNF-α and IL-6 among T2DM patients and found a positive correlation between the fasting blood glucose level and inflammation during diabetes (Buldak et al. 2012). Alolga et al. (2020) also clarified the relationship between inflammation and diabetes. Interfering the production of cytokines such as IL-6 and TNF-α by inhibiting the activation of the inflammation pathway may stop the progression of diabetes (Tsalamandris et al. 2019).

Siegesbeckiae Herba is a traditional herb that plays a pivotal role in the treatment of arthritis and rheumatism (Li et al. 2021). It is promising for its anti-inflammatory, antioxidative, and analgesic effects (Wang et al. 2011; Kim et al. 2014; Ren et al. 2020). Kirenol was recommended as an active compound that exerts antirheumatic and anti-inflammatory effects in Siegesbeckiae (Zhong et al. 2019). Kirenol could attenuate diabetic cardiomyopathy by regulating inflammation and fibrosis, and kirenol could inhibit the translocation of NF-κB and Smad2/3 from the cytoplasm to the nucleus and then suppress diabetic-induced cardio-inflammation and cardio-fibrosis (Wu et al. 2019). However, whether kirenol regulates the NF-κB signal pathway in diabetic kidney disease remains unknown. Here, diabetic mice showed activation of NF-κB and increased expression of IL-6 and TNF-α, we found kirenol could inhibit NF-κB activation as well as gene expression of IL-6 and TNF-α induced by diabetes.

We should mention that there are some limitations in this study, for example, the specific mechanism of how kirenol inhibited the TGF-β/Smads signal pathway and NF-κB signal pathway is still unclear, and further research is needed in the future.

## Conclusions

Taken together, for the first time, we found that kirenol could exert anti-inflammatory and anti-fibrotic effects in diabetic kidney disease via blocking both TGF-β/Smads and NF-κB signal pathways, as depicted in Figure 8. Our study provided a potential mechanism for the treatment of DN with kirenol. Although there are some limitations in this study, our findings indicated that kirenol might become a potential drug for the treatment of DN.
